# A rare case of isolated mitral valve endocarditis by *Gemella sanguinis:* Case report and review of the literature

**DOI:** 10.1016/j.ijscr.2020.03.001

**Published:** 2020-03-07

**Authors:** Antonios C. Sideris, Eric Zimmermann, Takuya Ogami, Dimitrios V. Avgerinos

**Affiliations:** a Thoracic Service, Deparment of Surgery, Memorial Sloan Kettering Cancer Center, 1275 York Av, New York, NY 10065, United States; b Department of Surgery, New York – Presbyterian Queens Hospital, Flushing, NY 11355, United States; c Department of Cardiothoracic Surgery, New York – Presbyterian Hospital, Weill Cornell Medical Center, New York, NY 10065, United States

**Keywords:** *Gemella sanguinis*, Endocarditis, Mitral valve, Case report

## Abstract

**Introduction:**

*Gemella sanguinis* is an extremely rare cause of infectious endocarditis, with only 12 cases previously reported in the literature. Here we report the third known case of isolated mitral valve endocarditis secondary to *G. sanguinis*.

**Presentation of case:**

A 53-year-old man with mitral valve prolapse and history of recent dental instrumentation presented with malaise, thigh and finger pain and new pansystolic murmur. He was diagnosed with severe mitral insufficiency due to infectious endocarditis secondary to *G. sanguinis*. He underwent mitral valve replacement and was treated with a long course of antibiotics.

**Discussion:**

*G. sanguinis* is a rare cause of infectious endocarditis with very few reported cases in the literature. In the majority of reported cases, a strategy of valve replacement along with prolonged antibiotic course results in good outcome for the patient.

## Highlights

• *Gemella sanguinis* is a very rare cause of infective endocarditis.
• Only 12 cases previously reported in the literature, only two of which involved exclusively the mitral valve.
• Usually associated with preexisting cardiac condition or oral infection/instrumentation.
• Valve replacement along with prolonged antibiotic course resulted in successful outcomes in the majority of cases.

## 1 Introduction

Infective endocarditis (IE) remains a potentially lethal condition with significant clinical impact. Gram positive cocci, including *Staphylococcus aureus*, viridans *streptococci* and *Enterococcus* spp. are the most common causative pathogens identified, followed by fungal and HACEK organisms [1]. *Gemella* spp. has been implicated as a rare IE pathogen. Here, we describe the third known case of isolated *Gemella sanguinis* mitral valve involvement. The patient was successfully treated with surgical valve replacement along with extended course of antibiotics. This work has been reported in accordance to the Surgical CAse REport (SCARE) guidelines [2].

## 2 Presentation of case

A 53-year-old male with known murmur for approximately 2 years presented to the emergency department of community-based tertiary care center with complaints of acute onset of right medial thigh pain associated with left toe and right fourth finger pain and swelling for 3 days that led to inability to move his leg. He denied any trauma to the area, fever, chills, chest pain or shortness of breath. He reported associated malaise, chills and anorexia for 4 months and 22 lbs. weight loss over 3 months for which he underwent a colonoscopy, CT scan of the chest, abdomen and pelvis and transthoracic echocardiogram which were reportedly unremarkable. He was diagnosed with mitral valve prolapse in Colombia about 3 months prior to presentation and one month later he underwent a root canal procedure for which he did not receive prophylactic antibiotics.

On physical exam, he was afebrile without conduction abnormalities, tachycardia or hypotension. A pansystolic murmur in the precordial area and tenderness in the right groin, right index finger and left big toe without evidence of acute limb or digit ischemia were present.

Laboratory workup revealed anemia with hemoglobin of 9.1 g/dL, leukocyte count 13,800 cells/mL with 82% neutrophils), normal platelet count (210,000 ells/mL), creatinine 1 mg/mL, CRP 14.5 mg/dL with ESR 98 mm/hr. CT of the abdomen and pelvis revealed hypodensity of the right medial hip and thigh corresponding to the area of patient's right hip pain.

A transthoracic echocardiogram showed normal left ventricular function, however the posterior mitral valve leaflet was flail with resulting eccentric, anteriorly directed severe mitral regurgitation. An echodensity on the posterior leaflet measuring 1.4 × 2.3 cm concerning for vegetation was appreciated. Three sets of blood cultures were obtained, and the patient was started empirically on vancomycin 1000 mg IV twice daily and ceftriaxone 2 g IV daily.

**Fig. 1:**
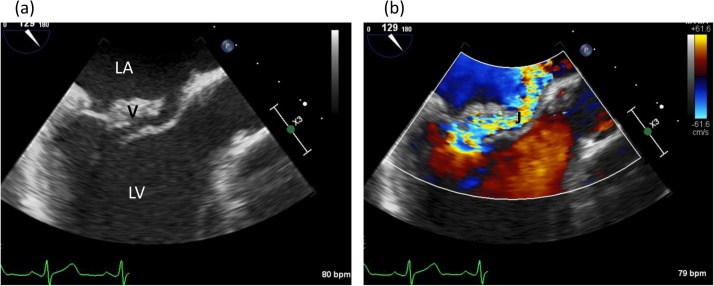
Parasternal long axis view showing vegetation on the posterior leaflet of the mitral valve (a). Doppler evaluation revealing severe mitral regurgitation (b). LA, left atrium; LV, left ventricle, Ao, aorta; V, vegetation; J, regurgitation jet.

A subsequent transesophageal echocardiogram confirmed the presence of severe MR with preserved LVEF and a large echodensity on the posterior leaflet measuring 1.3 × 0.7 cm, consistent with a vegetation (Fig. 1). Cardiac catheterization was negative for coronary artery disease. On right thigh MRI, prominent intramuscular edema throughout the right adductor muscles with a subtle focus of signal abnormality distally suspicious for a collection versus hematoma was found and was managed nonoperatively. Per the European Society of Cardiology (ESC) guidelines, the presence of subacute mitral regurgitation with unfavorable hemodynamic performance prompted us to offer the patient surgical intervention [3]. After discussing the risks and benefits of mechanical versus bioprosthetic device types, we followed the patient’s request to undergo open mitral valve replacement with a 31 mm St Jude Medical Epic Heart Valve (St Jude Medical, Inc, St Paul, MN). No attempt for mitral valve repair was made as there were multiple small anterior leaflet vegetations in addition to the large posterior leaflet vegetation.

His postoperative course was complicated by complete heart block, for which he underwent successful permanent pacemaker placement. His admission blood cultures grew *Gemella sanguinis* by VITEK 2 System (bioMérieux, Durham, NC), which was pan-sensitive on antibiogram. Vancomycin was switched to penicillin G 4,000,000 units every 6 h which was changed to daptomycin 8 mg/kg IV and ceftriaxone 2 g IV daily on discharge for a total of 6 weeks since valve replacement. The patient was seen in the office one month after his mitral valve replacement and he was making appropriate recovery.

## 3 Discussion and conclusion

*Gemella sanguinis*is a Gram-positive, non-spore forming, catalase-negative, oxidase-negative facultative anaerobe [4]. It is part of the normal oral, genitourinary and gastrointestinal flora and can be distinguished from the other *Gemella* spp. using biochemical tests and electrophoretic analysis of whole-cell protein [4,5].

A very small number of case reports of *G. sanguinis* IE have been published since its first isolation in 1998 [4,[6], [7], [8], [9], [10], [11], [12], [13], [14], [15], [16]]. Unlike most reported cases that involve the aortic valve, with or without additional native valve involvement, this is only the third reported case of isolated mitral valve involvement. Two prosthetic valve infections and an isolated tricuspid valve infection have been published. Preexisting cardiac and/or recent dental abnormalities were present in the majority of IE cases secondary to *Gemella* spp. (Table 1). Per ESC guidelines, antibiotic prophylaxis should be considered for dental procedures requiring manipulation of the gingival region, including such procedures as root canal as in the case of the current patient presentation (Class IIa, Level C evidence) [3].

**Table 1:** Summary of published studies of IE secondary to *Gemella sanguinis*.

Case	Year	Age/Gender	Cardiac Risk Factors	Oral Involvement	Valve	Antibiotic Regimen	Valve Replacement	Mortality
Collins et al.	1998	69 M	Unknown	Unknown	Unknown	Unknown	Unknown	Unknown
Shukla et al.	2002	69 M	No	Periodontal disease, tooth abscess	Mitral	Vancomycin + Gentamycin	Yes	No
Almaghrabi et al.	2009	23 F	Repaired ventricular septal defect, aortic valve regurgitation	Behçet disease (mouth ulcers)	Aortic	Ceftriaxone + Gentamycin	No	Yes
Gundre et al.	2011	26 F	Rheumatic heart disease, aortic/mitral valve replacement	Dental infection	Aortic - Prosthetic	Ceftriaxone	Yes	No
Yang et al.	2011	67 M	Rheumatic heart disease	Tooth infection	Aortic	Penicillin G	Yes	No
Tiu et al.	2012	27 F	Rheumatic heart disease, aortic/mitral valve replacement	Tooth infection	Aortic - Prosthetic	Ceftriaxone + Gentamycin	Yes	No
Rousseau-Gagnon et al.	2013	67 M	No	No	Aortic/Mitral/Tricuspid	Penicillin + Ceftriaxone	Yes	No
Chadha et al.	2013	73 M	No	No	Aortic/Mitral	Daptomycin + Gentamycin	Yes	No
Tsumita et al.	2015	57 F	No	No	Aortic	Vancomycin + Gentamycin	Yes	No
Mugunthan et al.	2016	4 M	No	No	Tricuspid	Vancomycin + Gentamycin	Yes	No
Emmanouilidou et al.	2019	85 F	Aortic regurgitation, mitral insufficiency	No	Mitral	Vancomycin + Gentamycin	No	No
Maraki et al.	2017	21 M	Bicuspid aortic valve	Recent dental cleaning, molar extraction	Aortic	Ceftriaxone + Gentamycin	Yes	No
Present Case	2019	53 M	Mitral valve prolapse	Recent root canal procedure	Mitral	Penicillin + Ceftriaxone	Yes	No

IE due to *G. sanguinis* appears to be relatively indolent and responds well to broad-spectrum antibiotics, including beta-lactams, vancomycin and/or aminoglycosides. Although successful non-operative management has been previously described [15], valve replacement has been required in the majority of cases. Surgical repair in conjunction with a prolonged course of IV antibiotics resulted in a good outcome, even in complicated cases.

Surgical replacement of the mitral valve was warranted for our patient secondary to the presence of severe mitral regurgitation with features of poor hemodynamic performance on presentation. Given the lack of robust data owing to the rarity of the infection, we would recommend that clinicians follow the current established recommendations for prophylaxis and surgical management of bacterial IE [3,17].

## Conflicts of interest

The authors report no conflicts of interest

## Funding

None

## Ethical approval

The New York Presbyterian – Queens IRB conducted a review of the submission and concluded that activities described in this study do not constitute human subjects research as the project does not involve identifiable private information from the patient and the subject has consented to the publication of their case. As a result, 455 CFR part 46 does not apply. A letter by Dr. Phyllis August, MD, MPH, Administrative Director of the New York Presbyterian – Queens IRB was provided and is available upon request.

## Consent

Written consent was obtained from the patient to publish a case report regarding his disease. It is available upon request.

## Author contribution

Antonios Sideris: Conceptualization, Investigation, writing – original draft, writing – review and editing; Takuya Ogami: writing – review and editing. Eric Zimmermann: writing – review and editing. Dimitrios Avgerinos: Conceptualization, supervision, writing – review and editing.

## Registration of research studies

N/A.

## Guarantor

The first and last author (ACS, DVA) accept full responsibility for the study and guarantee its accuracy.

## Provenance and peer review

Not commissioned, externally peer-reviewed.
